# The mediating effect of attitudes toward genetic testing on the relationship between genetic knowledge of familial hypercholesterolemia and the intention to undergo genetic testing among patients with hyperlipidemia in Korea: a cross-sectional study

**DOI:** 10.7586/jkbns.26.008

**Published:** 2026-08-28

**Authors:** Minji Kim, Myoungock Jang

**Affiliations:** 1 College of Nursing, Chungnam National University, Daejeon, Korea; 2 Chungnam National University Hospital, Daejeon, Korea

**Keywords:** Hyperlipoproteinemia type II, Health knowledge, attitudes, practice, Genetic testing, Intention

## Abstract

**Purpose:**

This study examined whether attitudes toward genetic testing mediated the relationship between genetic knowledge of familial hypercholesterolemia (FH) and intention to undergo genetic testing among patients with hyperlipidemia.

**Methods:**

This descriptive correlational, cross-sectional study included 140 adults with hyperlipidemia. Participants completed self-report questionnaires assessing genetic knowledge of FH, positive and negative attitudes toward genetic testing, and intention to undergo genetic testing. Data were analyzed using Pearson correlation analysis, multiple linear regression, and PROCESS Macro Model 4.

**Results:**

Multiple regression analysis showed that family history in a sibling (β = .20, *p* = .011), positive attitudes toward genetic testing (β = .41, *p* < .001), and negative attitudes toward genetic testing (β = -.24, *p* = .006) were significantly associated with the intention to undergo genetic testing. While the total effect of genetic knowledge on the intention was not significant (BootB = 0.04, 95% confidence interval [CI] [-0.001, 0.081]), positive attitudes toward genetic testing significantly mediated the relationship between genetic knowledge of FH and the intention to undergo genetic testing (direct effect = 0.03, *p* = .177; indirect effect = 0.01, 95% CI [0.001, 0.031]). Negative attitudes toward genetic testing did not mediate the relationship.

**Conclusion:**

Positive attitudes toward genetic testing were a significant mediator of the relationship between genetic knowledge of FH and intention to undergo genetic testing. These findings suggest that interventions intended to promote genetic testing should address patients’ positive attitudes toward testing rather than rely solely on information provision.

## INTRODUCTION

Hypercholesterolemia refers to an abnormal elevation of blood lipids or cholesterol levels, among which an increase in low-density lipoprotein cholesterol (LDL-C, “bad cholesterol”) constitutes a major risk factor for cardiovascular diseases [1]. The most common etiological factors include dietary patterns, sedentary lifestyles, and genetic predispositions, all of which contribute to the development of atherosclerotic plaque, vascular narrowing, and impaired blood flow [2]. Familial hypercholesterolemia (FH) represents a distinct subtype of hypercholesterolemia characterized by genetically driven, markedly elevated LDL-C concentrations, leading to a substantially increased lifetime risk of premature cardiovascular events [3].

FH is estimated to affect at least 1 in 500 individuals worldwide; however, recent European studies suggest a higher prevalence of approximately 1 in 250 [4]. In Korea, the prevalence appears comparable to the international rate, with an estimated 100,000 individuals living with FH [5]. Because patients with FH exhibit markedly elevated cholesterol levels from childhood, they have a substantially increased likelihood of developing acute coronary ischemic symptoms or myocardial infarction before the age of 30 [5]. Compared with the general population, individuals with FH also face higher risks of coronary artery disease, myocardial infarction, ischemic heart disease, and cerebrovascular disease [4,6]. A multinational cohort study including patients with FH from Denmark, the Netherlands, the United Kingdom, Japan, and Spain demonstrated that individuals receiving lipid-lowering therapy still had a 10.3-fold higher risk of cardiovascular disease compared with the general population, whereas untreated patients had an approximately 13.2-fold elevated risk [7]. These findings underscore the markedly heightened cardiovascular risk in FH and highlight that inadequate or absent treatment further exacerbates this burden [7]. Given these risks, early diagnosis through genetic testing is essential to ensure timely management, implement cascade screening for relatives, and reduce preventable cardiovascular morbidity and mortality [8].

For the diagnosis of FH, clinicians typically apply established diagnostic criteria that integrate physical examination findings, such as Achilles tendon xanthomas or corneal arcus presenting in childhood, family history of premature myocardial infarction before 50–60 years of age, serum cholesterol levels, and, when available, genetic testing [8]. However, in Korea, these characteristic physical manifestations are observed in only a subset of patients, underscoring the particular importance of genetic testing in confirming FH [9]. Current Korean recommendations suggest that, in adults with LDL-C levels > 190 mg/dL and a family history compatible with FH, genetic testing could be considered.

Experiences from other countries illustrate the potential benefits of implementing structured management strategies for FH. In the Netherlands, a nationwide FH genetic screening program from 1994 to 2014 integrated next-generation sequencing panels in order to substantially improve diagnostic accuracy [10]. Supported by government funding and collaboration with insurance providers, the program reduced patients’ financial burden and facilitated access to appropriate treatment. Through these coordinated efforts, approximately 40,000 individuals with FH were identified nationwide, with at least 1,500 new cases detected annually, enabling personalized and targeted treatment. Moreover, the program played a critical role in raising awareness of FH among both the general public and healthcare professionals, thereby strengthening early detection and long-term disease management.

Evidence on Korean patients diagnosed with FH remains relatively limited [9]. Awareness and understanding of FH are low not only among the general public but also among healthcare professionals, underscoring the need for early diagnosis and proactive management [9,11]. However, merely emphasizing the seriousness of the disease and the importance of genetic testing is insufficient to motivate patients to intend to undergo, or actually complete, genetic testing. An approach that focuses solely on risk information and the clinical necessity of testing does not adequately explain or promote individual behavior change [12]. Therefore, the application of a theoretical framework that can explain and predict an intention to undergo genetic testing is warranted.

The Theory of Planned Behavior (TPB) is a widely used framework for explaining and predicting health behaviors, positing that behavior is primarily determined by behavioral intention, which is the most proximal predictor of actual behavior [13,14]. Attitude toward the behavior, one of the key components of the TPB framework, is defined as an evaluation of the anticipated benefits and potential risks [13,14]. Although knowledge is not included as a core construct in the original TPB, it is widely incorporated to extend the application of the theory [15,16]. Before individuals can evaluate anticipated benefits and potential risks to form an attitude, they must first possess the relevant factual knowledge. In the context of FH, genetic knowledge may serve as the foundational informational input that shapes personal risk perception and behavioral beliefs. Therefore, knowledge contributes to behavioral intention primarily by shaping the evaluative process underlying attitude formation.

TPB has been applied and empirically supported in nursing and public health research to explain a variety of preventive and health-promoting behaviors, including vaccination uptake, cancer screening participation, and lifestyle modification [15]. Thus, the selective components of the TPB could provide an appropriate theoretical framework for understanding how knowledge about FH and attitudes toward genetic testing are translated into intention, and ultimately the planned uptake of genetic testing for FH [17]. This underscores the need to examine whether genetic knowledge is indirectly associated with the intention to undergo genetic testing by shaping attitudes toward genetic testing [18]. This study specifically focused on the attitude component informed by a previous meta-analysis suggesting that attitude is often the most robust predictor of behavioral intention in clinical preventive settings [19]. Therefore, drawing specifically on the attitude component of the TPB framework, this study aimed to examine the mediating role of attitudes toward genetic testing in the relationship between genetic knowledge of FH and the intention to undergo genetic testing among patients with hyperlipidemia.

## METHODS

### 1. Study design

This descriptive correlational cross-sectional study was conducted to examine the mediating role of attitudes toward genetic testing in the relationship between genetic knowledge of FH and the intention to undergo genetic testing among patients with hyperlipidemia. This study was conducted and reported in accordance with the Strengthening the Reporting of Observational Studies in Epidemiology guidelines [20].

### 2. Participants

The study was conducted at Chungnam National University Hospital located in Daejeon, South Korea and included patients diagnosed with hyperlipidemia. The inclusion criteria were as follows: adults aged 65 years or younger who (1) had at least one child; (2) had a diagnosis of hyperlipidemia alone, or had hyperlipidemia in combination with at least one of the following complications: coronary artery disease, myocardial infarction, ischemic heart disease, or other cardiovascular diseases; (3) had never undergone genetic testing or genetic counseling related to FH; and (4) were able to communicate and read Korean. Individuals with at least one child were targeted because a primary clinical utility of FH genetic testing lies in family-based cascade screening. Therefore, understanding the perspectives of patients with children is critical, as concerns about transmitting on a genetic condition may uniquely shape their attitudes and behavioral intentions.

The required sample size was calculated using G*Power 3.1.9.7 for multiple regression analysis. Because prior studies provided limited evidence regarding effect sizes for this topic, the sample size was calculated based on a medium effect size (*f*² = 0.15) as suggested by Cohen [21], a significance level of .05 and statistical power of 0.80. Assuming 12 predictors (i.e., age, sex, marital status, education level, number of children, monthly income, disease-related information seeking, age at diagnosis, family history, genetic knowledge, positive attitudes toward genetic testing, and negative attitudes toward genetic testing), the minimum required sample size for multiple linear regression analysis was estimated to be 127 participants. Considering a potential non-response rate of 10%, the target sample size was 140 patients.

### 3. Instruments

We collected data using a structured questionnaire composed of 43 items, including 14 items on general and disease-related characteristics, 13 items on genetic knowledge of FH, 15 items assessing positive and negative attitudes toward FH genetic testing, and one item measuring the intention to undergo genetic testing.

#### 1) General and disease-related characteristics

General characteristics included sociodemographic factors such as age, sex, marital status, education level, number of children, occupation, monthly income, and religion. Disease-related characteristics consisted of items assessing disease-related information sharing, information-seeking behaviors, duration since diagnosis, age at diagnosis, family history, and the presence of comorbid conditions.

#### 2) Genetic knowledge of FH

Genetic knowledge about FH was measured using items adapted and modified from the breast cancer knowledge tool developed by Choi et al. [22] and the Korean version of the Breast Cancer Genetic Counseling Knowledge Questionnaire [23,24]. The original items were modified by the researchers to reflect the clinical context of FH by replacing disease-specific terminology and aligning the genetic risk concepts with current FH guidelines. To ensure content equivalence while adapting the scales from breast cancer to FH, modifications were strictly limited to replacing disease-specific wording (e.g., replacing "breast cancer" with "FH"). The original instrument contained 15 items; however, two items (e.g., “A father can pass a breast cancer–related gene mutation to his daughter”) were excluded due to lack of relevance to the study population, resulting in a final set of 13 items. Face validity was then established by a nursing professor who reviewed the modified items for relevance and comprehensibility. Each item was answered as ‘yes,’ ‘no,’ or ‘don’t know.’ Correct responses were scored as 1, while incorrect or “don’t know” responses were scored as 0. Total scores ranged from 0 to 13, with higher scores indicating greater genetic knowledge. The reliability of the Korean version of the instrument reported by Park and Jun [24] was Cronbach’s alpha = .70. Cronbach’s alpha in the present study was .90.

#### 3) Attitudes toward genetic testing

Attitudes toward genetic testing were measured using a modified version of the Decisional Balance Scale for Breast Cancer Genetic Testing developed by Jacobsen et al. [25], which was translated and adapted for Korean breast cancer patients by Park and Jun [24]. For the present study, the items were further modified to reflect the context of FH genetic testing by replacing disease-specific wording (e.g., replacing "breast cancer" with "FH") and aligning genetic risk concepts with current FH guidelines. One item (i.e., Learning that I carry a genetic mutation may result in difficulties with insurance coverage or potential issues with employment), which was not applicable to this population, was removed, resulting in 15 items assessing positive attitudes (eight items) and negative attitudes (seven items). The measure comprised two subscales: positive attitudes, reflecting the anticipated benefits of genetic testing, and negative attitudes, reflecting the perceived risks and psychological burdens associated with genetic testing. Face validity was then established by a nursing professor who reviewed the modified items for relevance and comprehensibility. Items were rated on a 5-point Likert scale ranging from 1 (“strongly disagree”) to 5 (“strongly agree”). Park and Jun [24] reported Cronbach’s alpha values of .80 and .78 for the positive and negative subscales, respectively. In the present study, Cronbach’s alpha values were .84 and .72 for the positive and negative subscales, respectively.

#### 4) Intention to undergo genetic testing

The intention to undergo genetic testing was measured using a Korean-translated version of the intention to undergo genetic testing scale originally developed by Kessler et al. [26]. The instrument consisted of a single item asking participants about their likelihood of undergoing FH genetic testing, scored on a 4-point Likert scale: 1 (“I will not undergo genetic testing”), 2 (“I will consider genetic testing”), 3 (“I will probably undergo genetic testing”), and 4 (“I will definitely undergo genetic testing”). A single-item measure was selected because the intention to undergo genetic testing is a specific and unidimensional construct, making a single, direct assessment both practical and methodologically adequate for capturing the target behavior. Higher scores indicated a stronger intention to undergo genetic testing. The instrument was translated into Korean using a standard translation and back-translation procedure. Two bilingual researchers independently translated the items into Korean, discrepancies were reconciled, and another bilingual translator back-translated the reconciled version into English. The back-translated version was compared with the original to ensure equivalence, and further minor revisions were made.

### 4. Data collection

Before data collection, approval and permissions were obtained from the hospital’s nursing director and head nurses of the relevant wards. Participants were recruited from the cardiology outpatient clinic or the cardiology ward. The researcher provided a detailed explanation of the purpose and procedures of the study to all prospective participants, and informed consent was obtained. The researcher distributed the questionnaires directly to the participants, and the questionnaire required approximately 20 minutes to complete. Clinical characteristics were additionally obtained through a review of electronic medical records.

### 5. Data analysis

Data analysis was performed using SPSS version 29.0 (IBM Corp., Armonk, NY, USA). The following analytic procedures were performed: descriptive statistics (frequency, percentage, mean, and standard deviation) were used to summarize general characteristics, disease-related characteristics, FH genetic knowledge, positive and negative attitudes toward genetic testing, and the intention to undergo genetic testing. Group differences in the intention to undergo genetic testing according to participants’ general and disease-related characteristics were examined using t-tests and one-way analysis of variance, followed by Scheffé’s post-hoc test. After examination of bivariate relationships of the core variables, factors associated with the intention to undergo genetic testing were analyzed using multiple linear regression with the enter method. Variables for the multiple linear regression model were selected based on a combination of theoretical and statistical criteria. Specifically, the core variables of the study including genetic knowledge and positive and negative attitudes toward genetic testing were included based on the theoretical framework and bivariate relationships, while sociodemographic and clinical characteristics were included as covariates only if they demonstrated a statistically significant bivariate relationship with the intention to undergo genetic testing. Categorical variables, specifically sex and family history of hyperlipidemia in a sibling, were dummy-coded. Men and the absence of a family history in sibling served as the reference groups, respectively. Assumptions of normality, independence, homoscedasticity, and linearity were confirmed prior to analysis. Finally, the mediating effect of attitudes toward genetic testing on the relationship between genetic knowledge of FH and the intention to undergo genetic testing was evaluated using the PROCESS Macro (Model 4). Bootstrapped standard errors and confidence intervals for the indirect effect were estimated using 5,000 bootstrap resamples.

### 6. Ethical considerations

Approval from the Institutional Review Board (IRB No. 2024-08-008) of Chungnam National University Hospital in Daejeon, South Korea was obtained before conducting the study. Informed consent was obtained from all participants, and ethical standards were upheld throughout the study. Participants were informed of the study purpose, procedures, risks, and their rights, including voluntary participation, and anonymity. Participants also received an explanation assuring confidentiality and the exclusive use of their data for research purposes. They were informed of their right to refuse or withdraw from participation at any time. Participants were also assured that withdrawal would not negatively affect the quality of their healthcare. All completed questionnaires were stored in a locked cabinet accessible only to the research team.

## RESULTS

### 1. General and disease-related characteristics of participants

A total of 140 patients with hyperlipidemia participated in the study, and their general characteristics are presented in Table 1. The mean age was 56.96 years (standard deviation [SD] = 6.79, data not shown), and 45.0% were 60–65 years old, followed by 50–59 years (37.1%) and 30–49 years (17.9%). More than half of participants (64.3%) were men and the majority of participants were married (85.0%). Regarding occupation, the majority were employed (86.4%). In terms of education, 45.7% had completed college or higher, 39.3% had completed high school, and 15.0% had completed middle school or lower. More than half of the participants reported having a religion (51.4%). The most common number of children was two (61.4%).

Disease-related characteristics are also presented in Table 1. The internet was the most common source of disease information (48.6%), followed by healthcare professionals (38.6%). A family history of hyperlipidemia was reported by 40.7% of participants. Regarding the specific family members diagnosed, 18.6% reported only parents were affected, and 12.1% reported only siblings were affected, while 59.3% reported no family history or others. The age at diagnosis was most frequently 50–59 years (45.7%). For the duration since diagnosis, nearly half of the participants (48.6%) had been diagnosed for 60 months or more, while 34.3% had been diagnosed for 13 to 59 months. Most participants had at least one comorbidity, with 43.6% reporting one and 35.7% reporting two comorbid conditions.

### 2. Differences in the intention to undergo genetic testing by general and disease-related characteristics

Differences in the intention to undergo genetic testing according to participants’ general and disease-related characteristics are summarized in Table 1. Regarding age, there was a significant difference in the intention to undergo genetic testing (F = 3.26, *p* = .041). Post-hoc analysis revealed that participants aged 50–59 years had a significantly higher intention to undergo genetic testing than those aged 30–49 years. Women demonstrated a significantly higher intention to undergo genetic testing than men (t = -2.16, *p* = .033). Furthermore, there were significant group differences based on the family members diagnosed (F = 3.06, *p* = .019). Post-hoc analyses showed that individuals with only siblings diagnosed with hyperlipidemia had a significantly higher intention to undergo genetic testing compared to those with both parents and grandparents diagnosed. Other general and disease-related characteristics were not significantly associated with the intention to undergo genetic testing.

### 3. Levels of genetic knowledge, attitudes toward genetic testing, and intention to undergo genetic testing

Participants’ levels of genetic knowledge, positive and negative attitudes toward genetic testing, and the intention to undergo genetic testing are presented in Table 2. The mean score for genetic knowledge was 6.45 (SD = 3.59), with possible scores ranging from 0 to 13. Positive attitudes toward genetic testing, measured on a 5-point Likert scale, had a mean score of 4.01 (SD = 0.63), indicating a relatively positive tendency. Negative attitudes toward genetic testing had a mean score of 2.67 (SD = 0.68). The intention to undergo genetic testing, assessed on a 4-point scale, had a mean score of 2.71 (SD = 0.88), indicating a moderate level of intention.

### 4. Bivariate relationships of the variables

Bivariate relationship between core variables is presented in Appendix 1. Genetic knowledge was positively associated with positive attitudes toward genetic testing (r = .17, *p* = .049) and negatively associated with negative attitudes toward genetic testing (r = -.23, *p* = .006). However, genetic knowledge was not significantly associated with the intention to undergo genetic testing (r = .16, *p* = .054). Positive attitudes toward genetic testing were significantly associated with negative attitudes toward genetic testing (r = .41, *p* < .001) and the intention to undergo genetic testing (r = .34, *p* < .001). Finally, negative attitudes toward genetic testing were not significantly associated with the intention to undergo genetic testing (r = -.07, *p* = .417).

### 5. Factors associated with the intention to undergo genetic testing

Multiple linear regression analysis was conducted to identify factors associated with the intention to undergo genetic testing (Table 3). Prior to the analysis, the basic assumptions of the multiple regression model were examined and satisfied. The Durbin–Watson statistic was 1.87, supporting independence of residuals, and tolerance (0.73–0.93) and VIF values (1.07–1.37) indicated no multicollinearity concerns. The normality of residuals was confirmed by inspecting the normal P-P plot. Homoscedasticity and linearity were verified by observing a random, patternless distribution of points in the scatterplot of standardized residuals against standardized predicted values. Furthermore, an examination of standardized residuals (ranging from −2.35 to 1.95) and Cook's distance (maximum = 0.08) indicated no significant outliers or influential cases that could distort the model.

The regression model, which included six predictors (i.e., age, sex, family history in sibling, genetic knowledge, and positive and negative attitudes toward genetic testing) was statistically significant (F (6,133) = 7.11, *p* < .001), with an adjusted explanatory power (Adjusted *R*^2^) of 21%. Regarding the significant predictors, positive attitudes toward genetic testing emerged as the strongest predictor of the intention to undergo genetic testing (β =.41, *p* < .001). Negative attitudes toward genetic testing were negatively associated with the intention to undergo genetic testing (β = −.24, *p* = .006). Additionally, having a family history of hyperlipidemia in a sibling was positively associated with the intention to undergo genetic testing (β = .20, *p* = .011). In contrast, age (β = .04, *p* = .651), sex (β = .15, *p* = .057), and genetic knowledge (β = −.02, *p* = .856) were not significantly associated with the intention to undergo genetic testing.

### 6. Mediating effect of attitudes on the relationship between genetic knowledge and the intention to undergo genetic testing

**Figure 1.**
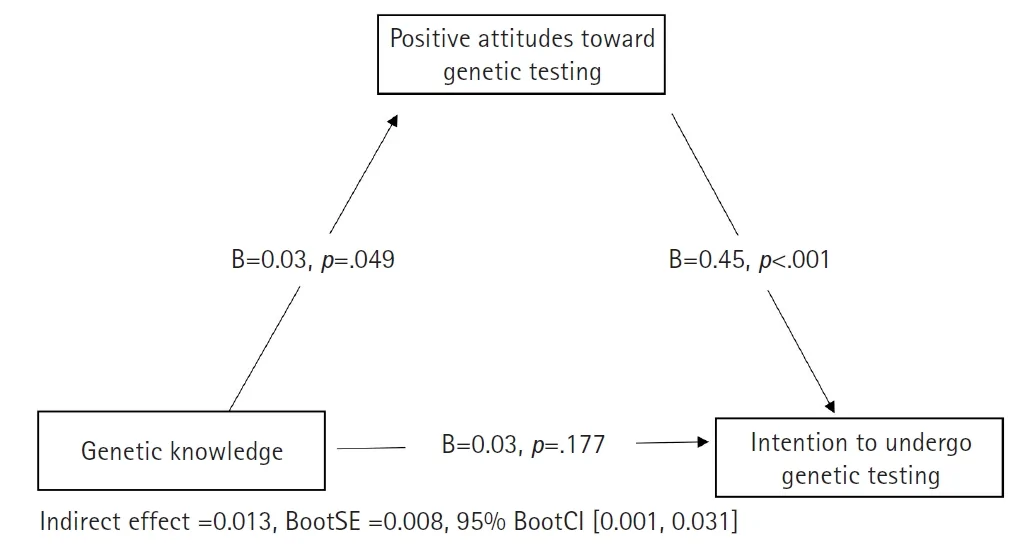
(A) Mediating role of positive attitudes toward genetic testing in the relationship between genetic knowledge and the intention to undergo genetic testing (N = 140). (B) Mediating role of negative attitudes toward genetic testing in the relationship between genetic knowledge and the intention to undergo genetic testing (N = 140). The path from genetic knowledge to intention represents the direct effect after inclusion of the mediator.

PROCESS Macro (Model 4) was employed to examine whether positive and negative attitudes toward genetic testing mediated the relationship between genetic knowledge and the intention to undergo genetic testing (Table 4). For Model 1, which focused on positive attitudes toward genetic testing, the direct effect of genetic knowledge on positive attitude toward genetic testing was statistically significant (B = 0.03, *p* = .049), whereas the direct relationship between genetic knowledge and the intention to undergo genetic testing was not significant (B = 0.03, *p* = .177). Positive attitudes toward genetic testing had a significant positive direct effect on the intention to undergo genetic testing (B = 0.45, *p* < .001). The total effect of genetic knowledge on the intention to undergo genetic testing was not significant (BootB = 0.04, 95% CI [-0.001, 0.081]). In terms of the mediating effect, positive attitudes toward genetic testing mediated the relationship between genetic knowledge and the intention to undergo genetic testing, as indicated by a significant indirect effect (BootB = 0.01, 95% CI [0.001, 0.031]) (Figure 1A).

For Model 2, which focused on negative attitudes toward genetic testing, the direct effect of genetic knowledge on negative attitude toward genetic testing was statistically significant (B = - 0.04, *p* = .006), whereas the direct effect of genetic knowledge on the intention to undergo genetic testing was not significant (B = 0.04, *p* = .074). Negative attitudes toward genetic testing did not have a significant direct effect on the intention to undergo genetic testing (B = -0.04, *p* = .706). The total effect of genetic knowledge on the intention to undergo genetic testing was not significant (BootB = 0.04, 95% CI [-0.001, 0.081]). With respect of the mediating effect, negative attitudes toward genetic testing did not mediate the relationship between genetic knowledge and the intention to undergo genetic testing, as indicated by a non-significant indirect effect (BootB = 0.002, 95% CI [-0.010, 0.013]) (Figure 1B).

## DISCUSSION

This descriptive cross-sectional study was conducted to examine the relationships among genetic knowledge of FH, positive and negative attitudes toward genetic testing, and the intention to undergo genetic testing among adults with hyperlipidemia who had no prior experience with genetic testing or counseling. Drawing upon the attitude component of the TPB, the study further assessed whether positive or negative attitudes toward genetic testing mediate the relationship between genetic knowledge of FH and the intention to undergo genetic testing. Our study results indicated that greater genetic knowledge was associated with more positive attitudes toward genetic testing, which, in turn, was associated with a higher intention to undergo genetic testing, thereby suggesting a significant indirect pathway. Several important findings emerged, offering insights into behavioral determinants of FH genetic testing within the Korean context.

The study participants demonstrated moderate levels of genetic knowledge, with considerable variability across individuals. This finding aligns with earlier reports suggesting that inadequate understanding of FH among both patients and healthcare professionals in Korea may contribute to its underdiagnosis and delayed treatment [9]. Moreover, prior research reporting extremely low use of FH diagnostic codes in clinical practice provided a structural basis for interpreting our findings, indicating that FH has yet to be adequately recognized or operationalized across the healthcare system [27]. In this context, a study reported that primary care physicians had a limited level of key FH-related knowledge [28]. Thus, limited genetic knowledge of the study participants may reflect not merely an individual-level deficit but also the combined influence of broader gaps in clinical awareness and constraints within health practices and systems. Considering that FH is a highly heritable condition requiring early identification and treatment, the generally moderate knowledge level observed in this study highlights the need for improved patient education and targeted communication strategies in clinical settings.

Our study participants showed relatively positive attitudes toward genetic testing. This finding is consistent with prior studies with the general population reporting overall positive attitudes toward genetic testing, particularly with respect to its preventive and health management value [29]. In contrast, the intention to undergo genetic testing was moderate, suggesting that recognition of its value does not necessarily translate into actual testing behavior due to psychological and environmental barriers [10]. These findings are consistent with evidence from a systematic review indicating that, in hereditary conditions such as FH, uptake of genetic testing and cascade screening remains low, largely due to psychological and financial burdens, limited accessibility, and emotional concerns about test results [30]. Additionally, because the current sample consisted of general patients with hyperlipidemia, it is likely that many did not strongly perceive themselves to be at specific genetic risk for FH, which may have further dampened the immediate intention to undergo genetic testing.

Importantly, the outcomes from multiple regression analysis indicated that a clinical variable (i.e., family history of hyperlipidemia) was a stronger predictor than baseline demographic differences like age and sex. Family history, especially the presence of an affected sibling, was significantly associated with a greater intention to undergo genetic testing. This finding is consistent with prior studies reporting that family history and familial disease experiences play an important role in FH risk perception and uptake of testing [31,32]. This study outcome suggests that family experiences of illness may make individuals’ risk perceptions more concrete and salient, thereby enhancing motivation to undergo genetic testing. Based upon our study’s findings, individuals with affected siblings may observe the clinical consequences of hyperlipidemia more closely, leading to heightened awareness and perceived personal risk. Although women and participants aged 50–59 years were likely to have a higher intention to undergo genetic testing in the unadjusted bivariate analysis, these differences did not retain statistical significance in the multiple regression model. These results suggest that health care providers should consider family dynamics and patterns of disease communication over general demographic characteristics when counseling patients about FH testing.

While positive attitudes toward genetic testing were significantly associated with the intention to undergo genetic testing, genetic knowledge lacked a significant direct association. Instead, genetic knowledge shaped the intention to undergo genetic testing through an indirect pathway by fostering positive attitudes toward genetic testing. This finding strongly supports our theoretical approach drawing upon the TPB framework, confirming that in the context of FH, knowledge functions as a distal antecedent providing the informational foundation, whereas attitude acts as the proximal evaluative construct that directly drives behavioral intention [13,14]. In other words, factual knowledge alone is insufficient to enhance intentions; instead, it serves as a foundational step that contributes to intention only insofar as it shapes an individual’s attitudes toward genetic testing [18,33].

Consequently, educational interventions should extend beyond the mere provision of factual risk information. Consistent with prior research on preventive health behaviors, clinical efforts must prioritize fostering positive attitudes by explicitly addressing the benefits, risks, and personal relevance of genetic testing, while also working to reduce psychological barriers [34]. Such a comprehensive approach may enhance the uptake of FH genetic testing, facilitate early detection, and ultimately contribute to the reduction of preventable cardiovascular events.

However, it is important to note that our regression model explained a relatively modest proportion of the variance in the intention to undergo genetic testing (21.0%). While capturing the attitude component provides valuable insights, it offers an incomplete picture compared to the full TPB framework, which typically explains a substantially larger proportion of variance (39%–40%) in health behavioral intentions [19], this model provides an incomplete picture. The intention to undergo genetic testing is likely associated with a broader array of external and systemic factors not captured in this model, such as perceived environmental barriers (e.g., cost and physical accessibility), psychological burdens like stigma, and explicit clinician recommendations.

Overall, our study findings suggest that the intention to undergo genetic testing among patients with hyperlipidemia is driven more by attitudes toward genetic testing than by genetic knowledge alone. From a theoretical perspective, this supports the view that, in the context of FH, knowledge functions as a distal antecedent providing the informational foundation, whereas the proximal evaluative construct (i.e., attitude) directly drives behavioral intention. Accordingly, interventions should prioritize fostering positive attitudes and reducing psychological barriers, rather than focusing solely on information provision. Such an approach may enhance the uptake of FH genetic testing, facilitate early detection, and ultimately contribute to the reduction of preventable cardiovascular events.

This study has several limitations. Although this study was guided by a well-established theoretical framework, its cross-sectional design limits the ability to infer causal relationships among the variables including genetic knowledge, positive and negative attitudes toward genetic test, and the intention to undergo genetic testing. Moreover, caution is warranted in generalizing the findings to the broader population because participants were recruited primarily from one healthcare institution in a limited geographic area. By drawing upon the attitude component of the TPB, we did not include other key constructs such as perceived behavioral control and subjective norms. The omission of these central components limits the overall predictive power of our model, as these unmeasured variables likely account for a substantial portion of the unexplained variance in intentions. In addition, because the analysis centered on individual knowledge and attitudes, external influences, such as perceived barriers to testing, practical accessibility, direct clinician recommendations, broader social perceptions, and the healthcare policy or system context, were not fully captured. Furthermore, the intention to undergo genetic testing was assessed using a single-item measure. While practical and directly capturing the target behavior, this approach limits the assessment of internal reliability and may reduce sensitivity and variance in regression and mediation analyses. Additionally, while the attitude toward genetic testing and genetic knowledge scales were adapted from validated breast cancer instruments and modified for FH, formal content validation processes (e.g., expert panel review) were not conducted. Furthermore, modifying and condensing the items in order to align with FH guidelines may have introduced item redundancy, which could explain the notably high internal consistency (Cronbach’s α = .90) observed in this study. Future research is warranted to formally validate this FH-specific attitude and knowledge scales to ensure its psychometric robustness.

Despite these limitations, this study has significant implications for practice, research, and policy. First, further research should extend the TPB-based model by incorporating other core components including subjective norms and perceived behavioral control of the TPB to more fully explain the relationships with the intention to undergo genetic testing. Longitudinal or prospective designs are also needed to test whether an improvement in FH-related knowledge contributes to sustained changes in attitudes and subsequent actual testing behavior. In addition, research may examine psychosocial mechanisms that may underlie attitudes (e.g., anticipated anxiety, stigma, perceived family responsibility, and perceived benefits/harms of results) and test mediators and moderators such as family history, prior family disease experiences, and clinician’s recommendation. Finally, intervention studies are warranted to evaluate whether attitude-focused counseling, family-centered education, and barrier-reduction strategies meaningfully increase cascade screening participation. The strong predictive power of attitudes in this study reinforces the need for intervention programs aimed specifically at enhancing positive attitudes, reducing misconceptions, and increasing perceived value of FH genetic testing.

For clinical practice, the findings suggest that genetic counseling and education for FH should move beyond delivering risk information and clinical necessity, and instead focus on shaping attitudes toward genetic testing and addressing emotional concerns that may inhibit action. The study outcome highlights the importance of structured genetic counseling, patient-friendly educational materials, and clinician communication strategies tailored to individuals with hyperlipidemia who may be unfamiliar with FH or genetic risk concepts. At the policy level, these findings highlight the need to strengthen system-wide recognition of FH by improving primary care capacity, standardizing diagnostic coding, and promoting structured cascade screening. Policies that reduce access barriers such as expanded reimbursement for genetic counseling and testing and streamlined service delivery may help translate awareness into uptake.

## CONCLUSION

Although the total effect of genetic knowledge on the intention to undergo genetic testing was not statistically significant, the findings demonstrate a statistically significant indirect association through positive attitudes toward genetic testing. These results highlight that improving patients’ genetic knowledge alone is insufficient; rather, shaping positive attitudes toward genetic testing is essential for increasing the uptake of genetic testing. FH remains underdiagnosed in Korea, and thus, these findings suggest that efforts to increase FH genetic testing uptake should go beyond providing risk information alone and instead focus on fostering positive attitudes toward genetic test, alleviating psychological burden, and incorporating family-based experiences and support. The results can serve as practical evidence to inform future FH genetic counseling strategies and the development of family-centered management programs.

## Figures and Tables

**Table 1.** Differences in the Intention to Undergo Genetic Testing by General and Disease-related Characteristics of Participants (N = 140)

Characteristics	Categories	n (%)	Mean (SD)	t/F(*p*) Scheffé
Age (years)	30–49^a^	25 (17.9)	2.32 (0.80)	3.26 (.041) b > a
	50–59^b^	52 (37.1)	2.85 (0.83)	
	60–65^c^	63 (45.0)	2.76 (0.93)	
Sex	Men	90 (64.3)	2.59 (0.81)	-2.16 (.033)
	Women	50 (35.7)	2.94 (0.98)	
Marital status	Married	119 (85.0)	2.71 (0.89)	0.34 (.714)
	Widowed	6 (4.3)	3.00 (0.89)	
	Divorced	15 (10.7)	2.67 (0.90)	
Employment	Yes (employed)	121 (86.4)	2.65 (0.82)	1.65 (.114)
	No (unemployed)	19 (13.6)	3.11 (1.15)	
Education level	Middle school or lower	21 (15.0)	2.95 (0.92)	1.45 (.238)
	High school	55 (39.3)	2.76 (0.94)	
	College or higher	64 (45.7)	2.59 (0.81)	
Religion	Yes	72 (51.4)	2.82 (0.88)	-1.45 (.148)
	No	68 (48.6)	2.60 (0.88)	
Number of children	1	38 (27.2)	2.55 (0.86)	1.81 (.168)
	2	86 (61.4)	2.63 (0.89)	
	3 or more	16 (11.4)	2.50 (0.89)	
Person to share health information	Spouse	93 (66.4)	2.68 (0.90)	0.66 (.683)
	Siblings	10 (7.1)	3.00 (0.94)	
	Parents	10 (7.1)	2.50 (0.85)	
	Children	9 (6.4)	2.89 (0.78)	
	Friends	5 (3.6)	3.20 (1.10)	
	Healthcare providers	6 (4.3)	2.50 (1.04)	
	No one	7 (5.1)	2.71 (0.95)	
Health information seeking method	Internet	68 (48.6)	2.68 (0.91)	0.98 (.423)
	TV/Radio	11 (7.8)	2.64 (0.28)	
	Friends	6 (4.3)	2.83 (0.41)	
	Fellow patients	1 (0.7)	4.00 (0.00)	
	Healthcare providers	54 (38.6)	2.74 (0.83)	
Family history of hyperlipidemia	Yes	57 (40.7)	2.89 (0.90)	2.24 (.110)
	No	57 (40.7)	2.63 (0.79)	
	Not sure	26 (18.6)	2.62 (0.99)	
Family members diagnosed	Parents only^a^	26 (18.6)	2.62 (0.75)	3.06 (.019) b > e
	Siblings only^b^	17 (12.1)	3.35 (0.93)	
	Children only^c^	2 (1.4)	3.00 (1.41)	
	Parents + Siblings^d^	11 (7.9)	2.91 (0.94)	
	Parents + Grandparents^e^	1 (0.7)	2.00 (0.00)	
	None/others^f^	83 (59.3)	2.59 (0.86)	
Age at diagnosis (years)	30–39	10 (7.1)	2.30 (0.48)	0.93 (.428)
	40–49	39 (27.9)	2.54 (0.85)	
	50–59	64 (45.7)	2.83 (0.91)	
	60–65	27 (19.3)	2.78 (0.63)	
Duration since diagnosis (months)	1–12	24 (17.1)	2.71 (0.96)	0.00 (.997)
	13–59	48 (34.3)	2.71 (0.90)	
	≥ 60	68 (48.6)	2.72 (0.86)	
Presence of comorbidities	None	9 (6.4)	2.89 (0.78)	1.21 (.310)
	One	61 (43.6)	2.70 (0.90)	
	Two	50 (35.7)	2.82 (0.89)	
	Three or more	20 (14.3)	2.40 (0.82)	

SD = Standard deviation.

**Table 2.** Levels of Genetic Knowledge, Positive Attitudes, Negative Attitudes, and Intention to Undergo Genetic Testing among Participants (N = 140)

Variables	Mean (SD)	Minimum	Maximum	Range
Genetic knowledge	6.45 (3.59)	0	13.0	0–13
Positive attitudes toward genetic testing	4.01 (0.63)	1.0	5.0	1–5
Negative attitudes toward genetic testing	2.67 (0.68)	1.0	4.7	1–5
Intention to undergo genetic testing	2.71 (0.88)	1.0	4.0	1–4

SD = Standard deviation.

**Table 3.** Factors Associated with Intention to Undergo Genetic Testing (N = 140)

Variables	B	SE	β	t(*p*)	VIF
Age	0.01	0.01	.04	0.45 (.651)	1.08
Sex (ref. = men)	0.28	0.14	.15	1.92 (.057)	1.08
Family history of hyperlipidemia (ref. = no family history in sibling)	0.54	0.21	.20	2.59 (.011)	1.07
Genetic knowledge	–0.00	0.02	-.02	–0.18 (.856)	1.22
Positive attitudes toward genetic testing	0.58	0.12	.41	4.67 (<.001)	1.37
Negative attitudes toward genetic testing	–0.32	0.12	–.24	–2.77 (.006)	1.37
R²	.24				
Adjusted R²	.21				
F(6, 133)	7.11				
*p*	< .001				

Reference group: sex (men); family history of hyperlipidemia in sibling (siblings only + parents & siblings, Yes=1, No=0); Durbin-Watson = 1.87; collinearity tolerance = 0.73-0.93. SE = Standard error; VIF = Variance inflation factor.

**Table 4.** Direct, Total, and Indirect Effects of Genetic Knowledge on Intention to Undergo Genetic Testing through Positive and Negative Attitudes toward Genetic Testing (N = 140)

Model 1	B	β	SE	t(*p*)	R²	adj. R²
Direct effect						
Genetic knowledge → Positive attitude	0.03	.17	0.01	1.99 (.049)	.03	.02
Genetic knowledge → Intention	0.03	.11	0.02	1.36 (.177)	.13	.11
Positive attitude → Intention	0.45	.32	0.11	3.97 (<.001)		
	BootB		BootSE	t(p)	95% BootCI	
					LL	UL
Total effect						
Genetic knowledge → Intention	0.04	—	0.02	1.95 (.054)	–0.001	0.081
Indirect effect						
Genetic knowledge → Positive attitude →Intention	0.01	.05	0.01	—	0.001	0.031
**Model 2**	**B**	**β**	**SE**	**t(p)**	**R²**	**adj. R²**
Direct effect						
Genetic knowledge → Negative attitude	–0.04	–.23	0.02	–2.82(.006)	.06	.05
Genetic knowledge → Intention	0.04	.16	0.02	1.80(.074)	.03	.01
Negative attitude → Intention	–0.04	–.03	0.11	–0.38(.706)		
	BootB		BootSE	t(p)	95% BootCI	
					LL	UL
Total effect						
Genetic knowledge → Intention	0.04	—	0.02	1.95 (.054)	–0.001	0.081
Indirect effect						
Genetic Knowledge → Negative attitude → Intention	0.00	.01	0.01	—	–0.010	0.013

BootSE and BootCI for the indirect effect were estimated using bootstrapping with 5,000 resamples. SE = Standard error; CI = Confidence interval.

## Appendix 1. Pearson Correlation Coefficients among Genetic Knowledge, Positive Attitudes, Negative Attitudes, and Intention to Undergo Genetic Testing (N = 140)

**Table:**

Variables	1	2	3	4
	r (*p*)			
1	1	-	-	-
2	.17 (.049)	1	-	-
3	-.23 (.006)	.41 (< .001)	1	-
4	.16 (.054)	.34 (< .001)	-.07 (.417)	1

1 = Genetic knowledge, 2 = Positive attitude, 3 = Negative attitude, 4 = Genetic testing intention.
